# Distinctive Characters of Remittent, Typhoid, and Typhus Fevers

**Published:** 1846-04

**Authors:** Austin Flint


13. *Distinctive characters of Remittent, Typhoid, and Typhus Fevers.* By Austin Flint, M.D.—Nosological classifications of fevers, and the nomenclature of this class of diseases, have been as mutable as the numerous theories respecting the intimate pathological perversions constituting the febrile state. *Inflammatory*, *putrid*, *adynamic*, *bilious*, *yellow*, *jail*, *nervous*, *malignant*, *petechial*, *congestive*, *ataxic*, *gastro-enterite*, and a host of other names have been at different times, and by different writers, employed to distinguish forms of this disease, their application being based upon circumstances ascertained or imagined, appertaining to their origin, location, peculiar complications, or visible appearances. It is easy to raise objections to each of these appellations, and, indeed, so long as the essential pathology of the febrile state remains unknown, so long will any arrangement and nomenclature be liable to be overturned and rendered obsolete by the ever changing results of progressive, pathological investigations. The distinction between periodic and continued fevers, being founded on obvious facts, has been retained for a long time, and will probably be permanent; but the different varieties of the latter are still subjects of discussion, and the occasion for much difference of doctrine. The term *typhus* has been in use ever since the time of Hippocrates, to denote a variety of fever characterized by peculiar conditions of the mind and general powers, which are expressed in its etymology. From its appropriateness and long use, it is not probable that this term will be soon abandoned; moreover, modern investigations have shown that the variety of the disease which it is intended to express is very distinctively marked, not only by the cerebral symptoms and general prostration, but by other and peculiar phenomena. Heretofore its application was not so well defined as it is now. By Armstrong, whose writings until lately were deemed of the highest authority, its latitude was extended so as to embrace, by means of qualifying adjectives, a great proportion of the cases of continued fever. It has now become more limited, embracing only a variety distinguished by peculiar symptoms, attended by a constant exanthema, and especially involving, as its remote cause, (in addition to contagion or infection) concentrated animal effluvia. It is exceedingly rare that an opportunity presents itself for witnessing specimens of true Typhus in this locality. It has fallen under the writer's observation in two well marked instances, only, during the last ten years. In one of these it occurred in the family of an Irish emigrant, who had recently arrived in this country. One of three children had been affected and died with it on their passage. The other two were attacked on their arrival in this place. The other instance occurred at the alms house near this city in the winter of 1840-41. Four cases transpired there in quick succession, under circumstances which rendered it almost certain that the origin of the disease was due to the malaria engendered by close congregation of the inmates of the house in unventilated apartments. These cases were reported for the Boston Med. and Surg. Journal, June, 1841.

The term *typhoid*, as applied to distinguish a certain class of fevers, is of recent date, and is not recognized in this sense by all pathologists. It originated with Louis, of Paris, whose researches into the history and comparative phenomena of the fevers of the Parisian hospitals, may be said to have created a new era for this department of pathological science, and will remain an imperishable monument of unsparing industry, and professional zeal. The investigations of this truth loving, talented pathologist, in themselves of great value, are still more valuable as furnishing a new point of departure, and giving impulse to a movement, which has enlisted the efforts of a multitude of co-laborers in different countries. Louis ascertained that the fevers of Paris were allied to each other by a certain uniformity of lesions, the most constant of which were disorganization of the glands of Peyer, and a softened condition of the spleen. The same disease, by those who believe it to be in fact a local affection, of which the febrile symptoms are symptomatic, has been entitled *Dothinenterite* or *follicular enteritis*. After these minute and comprehensive investigations in France, it became an interesting and important point of inquiry, in how far the same form of fever prevails in other countries. The attention of pathologists was soon directed to this point. In the British Islands it was found that in some of the cases of the, there so called, typhus fever, the descriptions of Louis were applicable, and in others they were not. Thence, it appears to be the prevailing opinion of British writers that the typhus and typhoid forms of fever are identical, but presented under different modifications. They do not generally acknowledge typhoid fever as a distinct form of the disease. In our country it has been ascertained by Jackson, Hale, Shattuck, and others of Boston, that the fever indigenous in New England, and known there as the common-continued, or autumnal fever, presents, in the phenomena before and after death, an entire similarity to the typhoid fever as portrayed by Louis. Gerhard, of Philadelphia, has also described a fever indigenous in that locality, identical with the typhoid; and, also, an epidemic typhus, which he traced to importation from England by means of English immigrants arriving there, in which the characteristic symptoms and lesions of typhoid fever were absent. He regards the two forms of fever as distinctly separable; and having had abundant opportunities to study the disease as it presents itself abroad and at home, his opinions are entitled to great consideration. Nevertheless, it is by no means regarded as settled by pathologists of this country, that the two forms of disease are intrinsically distinct. It is still a question *sub-judice* whether they are not one and the same disease, presented at different times and places under different modifications. While there is much weight of authority for the negative of this question, the numerical vote, we imagine, would be in favor of the affirmative.

We have extended these remarks farther than we intended when we commenced writing. Without entering into the controversial question just referred to, it had occurred to us that it would be acceptable to our readers to present a brief synopsis of the distinctive characters appertaining to the forms of fever distinguished as Typhus and Typhoid, and also those which belong to the common Remittent fever, as distinguished from both of the preceding. Whether we accept the doctrine which makes a specific distinction between the two forms, or that which regards them as varieties of the same species, there can be no doubt that the distinctions exist, and it is, to say the least, convenient to indicate this fact by different terms. The fever which generally prevails in this locality, is the common remittent, or, as it is miscalled, *bilious* fever. But there is reason to suppose that sporadic cases of typhoid fever occasionally occur, and we are liable, at any time, to meet with this or undoubted typhus in an epidemic form. Under these circumstances, it appears to us that there will be an advantage in having a synoptical view of these several forms of the disease which will admit of easy reference.

### CHARACTERS DISTINGUISHING REMITTENT & TYPHOID FEVER.

*Typhoid.*—Access is gradual: a week or more with prodromic symptoms.

*Remittent.*—Access is generally sudden.

*Typhoid.*—Characterized by prostration and stupor ("typhoid symptoms") early in the disease.

*Remittent.*—The symptoms commonly called "typhoid," do not occur until quite late in the disease.

*Typhoid.*—Seldom a distinct chill, but only chilly sensations, which do not occur with periodicity.

*Remittent.*—Chills generally produced, and apt to recur several times with observance of periodicity.

*Typhoid.*—Diarrhœa one of the most constant symptoms; occurs early, and is apt to continue. Meteorism is generally present.

*Remittent.*—Diarrhœa only an occasional symptom. Meteorism of less frequent occurrence.

*Typhoid.*—Sordes of mouth, with low muttering delirium early in the disease.

*Remittent.*—These symptoms are not found until the latter part of the disease, and very often absent.

*Typhoid.*—Attacks subjects, almost invariably, under 30 years of age.

*Remittent.*—Shows no such discrimination.

*Typhoid.*—Cough and Expectoration (Bronchitis) are generally prominent symptoms.

*Remittent* —Of much less frequency.

*Typhoid.*—Rose, or red spots *(taches rouges)* occur over abdomen and breast, from the 8th to 15th day.

*Remittent.*—Absent.

*Typhoid.*—Tongue is brown and dark.

*Remittent.*—Generally yellowish white. Nausea and vomiting are characteristic of its early stage; while they are less prominent, and often absent in typhoid. The conjunctiva is frequently yellow, and the urine impregnated with bile. The skin is apt to be yellow or sallow. These symptoms do not occur in typhoid. The mind, also, in remittents, is frequently unaffected; very rarely so in typhoid.

*Typhoid.*—Subsultus frequently present.

*Remittent.*—Rarely.

*Typhoid.*—Occurs rarely before November, sometimes in the latter part of October.

*Remittent.*—Occurs especially in August and September; unfrequent in October, and rarely afterward.

*Typhoid.*—Contagious and infectious.

*Remittent.*—No evidence that it is either.

*Typhoid.*—Presents after death disease of the Elliptical Plates, or Peyers glands, the mesenteric ganglions, and softening of spleen.

*Remittent.*—Spleen may be softened. The other lesions mentioned, rare. Stomach and Liver almost invariably evince disease. (Stewardson.) A fact which often enables us to confirm our diagnosis of remittent fever at its close, or after the case has terminated, is this: It is very apt to resolve itself into an intermittent form; and, in a very large proportion of cases, individuals who have had remittent fever, experience an attack of intermittent within a twelve month after their recovery. This fact may, also, serve to correct a diagnosis of typhoid fever if it has been incorrectly made.

### CHARACTERS DISTINGUISHING TYPHUS AND TYPHOID FEVER.

The following is an abstract of the diagnostic symptoms distinguishing the two forms of fever above mentioned, presented by Dr. Gerhard, in his lecture on the subject, published in connection with the lectures of Dr. Graves, of Dublin. The descriptions are abbreviated as much as practicable. He classes the symptoms under four heads.

#### CEREBRAL AND NERVOUS SYMPTOMS.

*Typhoid.*—Loss of strength and prostration very early in the disease. Singing in ears, vertigo, epistaxis. Pains in head and limbs not so violent as in intermittent and remittent fevers. Chillness, but not defined chill. Brain symptoms increase slowly. Delirium rarely entirely wanting; sometimes only at night.

*Typhus.*—Stupor prominent from the first. Patient becomes comatose at an early period. Delirium always of the still, muttering kind.

#### OF THE SKIN.

*Typhoid.*—Rose spots fewer in number, often only six or eight; rarely more than thirty; rather larger, elliptical, and more elevated.

*Typhus.*—Exanthema extends over the whole body. Papulæ are rounded, varying in size from an imperceptible point to breadth of a line. Occurs on the 3d day. May continue 12 or 14 days—generally only 5.

ABDOMINAL ORGANS.

*Typhoid.*—Diarrhœa after a few days, a frequent, but not invariable symptom. Meteorism, or abdominal pains generally present.

*Typhus.*—These symptoms are not present excepting as rare and accidental complications. Thirst more marked.

THORACIC SYMPTOMS.

*Typhoid.*—Some congestion of smaller bronchial tubes, which may pass into bronchitis, or pneumonitis.

*Typhus.*—Congestion different: apparently more dependent on the state of the blood, which obeys the laws of gravitation —the dependent part always full of blood. Pulse more frequent. Eye more dull, heavy, and blood-shot.

He states the following general circumstances:

Typhus spares no age; prevails as an epidemic, extending by contagion, or direct propagation from an infected individual.

Typhoid rarely assumes this character; rarely epidemic, and scarcely infectious, *excepting when prevailing epidemically.*

The medical physiognomy is very diagnostic, but difficult to be described.

To these it is to be added that the characteristic intestinal lesion of typhoid fever, or, to use the language of Louis, its anatomical character, while it is essential to constitute this disease, is an infrequent complication only in typhus.

In view of the acknowledged ability of Dr. Gerhard as a pathologist, his extensive and peculiar opportunities for clinical observation, and the fact that he has advocated the doctrine of an essential distinction between these two forms of fever, it seems fair to conclude that he would submit as strong a contrast of diagnostic symptoms as will comport with a rigid adherence to an exact description of phenomena. The practical reader will perhaps be enabled, from the preceding comparison, to form an opinion whether the antithetic characters are sufficient to constitute a radical distinction, or whether they are not of so kindred a nature as to denote the same disease under somewhat different aspects. For our own part we incline to the latter view, although we admit that the circumstances indicated, enable the practitioner to determine in most cases, without much trouble, whether the disease be more appropriately—agreeably to this classification—called typhus or typhoid; and that it is convenient and useful to make the distinction. In short, while all must acknowledge that they are in nearness of kin,—cousin-germans—we are disposed to regard them as even more closely allied to each other, being, if not one and the same individual, at least pathological twin-brothers.—*Buff. Med. Jour.*, January, 1846.

*November*, 1845.

